# Supplementary material for: Salmonella Surveillance Systems in Swine and Humans in Spain: A Review
Source: Vet Sci. 2019 Feb 20;6(1):20. doi: 10.3390/vetsci6010020 (PMC6466228; doi:10.3390/vetsci6010020)
Supplement: Supplementary file 1 [file vetsci-06-00020-s001.pdf]

veterinary sciences

MDPI

Article

Supplementary Materials: *Salmonella* Surveillance Systems in Swine and Humans in Spain: A Review

Marta Martínez-Avilés [1,]*, Macarena Garrido-Estepa [1], Julio Álvarez [2,3] and Ana de la Torre [1]

1 Animal Health Research Center (INIA-CISA), Ctra Algete a El Casar s/n, 28130, Valdeolmos, Madrid,28040, Spain; garrido.macarena@inia.es
2 VISAVET Animal Health Surveillance Center, Complutense University of Madrid, Madrid, 28040, Spain; jalvarez@visavet.ucm.es
3 Animal Health Department, Veterinary School, Complutense University of Madrid, Madrid, 28040, Spain; torre@inia.es
* Correspondence: martinez.marta@inia.es; Tel.: +34-91-620-2300

Received: 10 January 2019; Accepted: 16 February 2019; Published: date

Comentado [MDPI1]: Please carefully check the accuracy of names and affiliations.

Comentado [MDPI2]: Do you have department for the institution?

Comentado [MDPI3]: Please provide full name.

Table S1. Annex 1. Literature search results with the terms "s*Salmonella*", "pigs", "Spain", "epidemiology".

Comentado [MDPI4]: Please confirm the title.

Con formato: Fuente: Cursiva

	AUTHORS	TITLE	TYPE	YEAR	REFERENCE
**1**	Mejía, W., et al.	Epidemiology of *Salmonella* infections in pig units and antimicrobial susceptibility profiles of the strains of *Salmonella* species isolated	Scientific article	2006	Veterinary Record, 159: 271-276
**2**	García Feliz, C., et al.	*Salmonella enterica* Infections in Spanish Swine Fattening Units	Scientific article	2007	Zoonoses and Public Health 54: 294-300
**3**	García Feliz, C., et al.	Antimicrobial Resistance of *Salmonella enterica* Isolates from Apparently Healthy and Clinically Ill Finishing Pigs in Spain	Scientific article	2008	Zoonoses and Public Health, 55: 195-205
**4**	Vico, J.P., et al.	Evaluation of three commercial enzyme-linked immunosorbent assays for the detection of antibodies against *Salmonella spp.* in meat juice from finishing pigs in Spain	Scientific article	2010	Zoonoses and Public Health 57 (Suppl.1):107-114
**5**	García Feliz, C.	Swine salmonellosis in Spain: prevalence, risk factors and antimicrobial resistance [in Spanish]	PhD Thesis	2011	León University, Spain, 211 pp.
**6**	Gómez-Laguna, J., et al.	Prevalence and antimicrobial susceptibility of *Salmonella* infections in free-range pigs	Scientific article	2011	The Veterinary Journal, 190: 176-78
**7**	Vico, J.P., et al.	Salmonellosis in Finishing Pigs in Spain: Prevalence, Antimicrobial Agent Susceptibilities, and Risk Factor Analysis	Scientific article	2011	Journal of Food Protection, 74 (7): 1070-1078

**8**	Andrés, S., et al.	Epidemiology of Subclinical Salmonellosis in Wild Birds from an Area of High Prevalence of Pig Salmonellosis: Phenotypic and Genetic Profiles of *Salmonella* Isolates	Scientific article	2013	Zoonoses and Public Health, 60: 355-365
**9**	Argüello Rodríguez, H.	Swine salmonellosis in Spain: risk factors in breeding pigs, control strategy in fattening pigs and importance at slaughter [in Spanish]	PhD Thesis	2013	León University, Spain, 331 pp.
**10**	Argüello, H., et al.	Sero- and genotyping of *Salmonella* in slaughter pigs, from farm to cutting plant, with a focus on the slaughter process	Scientific article	2013	International Journal of Food Microbiology, 161: 44-52
**11**	Andrés-Barranco, S., et al.	Role of Wild Bird and Rodents in the Epidemiology of Subclinical Salmonellosis in Finishing Pigs	Scientific article	2014	Foodborne Pathogens and Disease, 11 (9): 689-697
**12**	González Clari, M.	Epidemiology of *Salmonella spp.* In fattening pigs [in Spanish]	PhD Thesis	2014	Cardenal Herrera University- CEU, Spain, 248 pp.
**13**	Casanova-Higes, A., et al.	Influence of on-farm pig *Salmonella* status on *Salmonella* shedding at slaughter	Scientific article	2017	Zoonoses and Public Health 2017, 64: 328-336
**14**	Mainar-Jaime, R., et al.	*Salmonella enterica* Infections in Spanish Swine Fattening Units	Scientific article	2018	Zoonoses and Public Health 65:e222-e228
